# Research progress on combat trauma treatment in cold regions

**DOI:** 10.1186/2054-9369-1-8

**Published:** 2014-05-01

**Authors:** Hui-Shan Wang, Jin-Song Han

**Affiliations:** Department of Cardiovascular Surgery, General Hospital of Shenyang Military Command, Shenyang, 110016 China

**Keywords:** Military medicine, Wounds and injuries, Cold climate, Body temperature

## Abstract

Cold regions are a special combat environment in which low temperatures have a great impact on human metabolism and other vital functions, including the nervous, motion, cardiovascular, circulatory, respiratory, and urinary systems; consequently, low temperatures often aggravate existing trauma, leading to high mortality rates if rapid and appropriate treatment is not provided. Hypothermia is an independent risk factor of fatality following combat trauma; therefore, proactive preventative measures are needed to reduce the rate of mortality. After summarizing the basic research on battlefield environments and progress in the prevention and treatment of trauma, this article concludes that current treatment and prevention measures for combat trauma in cold regions are inadequate. Future molecular biology studies are needed to elucidate the mechanisms and relevant cell factors underlying bodily injury caused by cold environment, a research goal will also allow further exploration of corresponding treatments.

Atypical battlefield environments often differ from more common environments in combat trauma pattern, injury type, and the applied principles of treatment. Cold regions are a distinct type of battlefield environment that feature lengthy periods of extreme temperature differences, harsh conditions, multiple disease factors, various injury types, and a wide distribution of frozen soil, snow, ice, and complex terrain. The high impact of environmental factors (e.g., low temperature) often aggravates the original wound and may result in a higher mortality rate if suitable treatment is not rapidly applied. Furthermore, victim evacuation is often difficult, thereby significantly increasing both combat and non-combat casualties. In the Korean War, low temperature-induced non-combat attrition accounted for 20% of the total casualties in the People’s Volunteer Army. Therefore, combat trauma treatment in cold regions is a key issue for military medicine research in the People’s Liberation Army (PLA). Here, we reviewed the worldwide research progress of combat trauma treatment in cold regions.

## Basic research on the influence of cold environments on the body

The most common source of temperature loss in trauma patients includes: exposure (environmental, as well as cavitary), the administration of i.v. fluids, and anesthesia which results in the loss of shivering mechanisms and decreases blood pressure [1]. The normal range in human body temperature is generally considered to be between 36.4-37.3°C, and non-traumatic hypothermia is defined at a core body temperature between 32-35°C. If injuries are involved, hypothermia is defined by a core body temperature below 35°C. Mestre-Alfaro *et al.*[2] proposed that the human body is less adaptive to extreme cold than to high temperatures; low temperatures can cause frostbite, severely damage the cardiovascular, respiratory, urinary, immune and motor systems, and may even be life-threatening.

## The influence of a cold environment on the central nervous system

A short stimulation of sympathetic nerves by a cold environment can enhance nerve tension and metabolic activity. With prolonged cold exposure, human motor and sensory neurons are also functionally suppressed, which leads to numbness and irreversible damages [3]. Kühlein *et al.*[4] experimentally demonstrated that cold injury can slow cranial nerve transduction and may indirectly intensify cold injury via oxidative damage, inducing brain edema, secondary brain injury, and apoptosis. Every 1°C reduction in body temperature corresponds to a decrease in cerebral blood flow by 6%-7%, which can endanger the lives of patients sustaining traumatic brain injury.

## The influence of cold environment on the motor system

Blondin and colleagues [5]. revealed that cold exposure can boost aerobic oxidation and energy metabolism in skeletal muscle, resulting in elevated heat generation to sustain body temperature. In addition, exposure to cold may cause dysfunction in the peripheral nervous system and correspondingly impair skin sensation, diminish muscle coordination, and decrease joint flexibility. Together, these impairments can result in torn muscles and tendons.

## The influence of cold environment on the cardiovascular system

The early effects of hypothermia on the cardiovascular system include augmented heart rate, output, and mean arterial pressure. However, with continuing decrease in body temperature, other symptoms begin to emerge such as slowed heart rate, weak myocardial contractility, reduced output, and low blood pressure [6]. When body temperature drops below 33°C, coronary blood flow begins to diminish, resulting in myocardial anoxia. Atrial fibrillation arises when body temperature falls below 30°C, and ventricular fibrillation occurs when body temperature falls below 25°C; cardiac arrest develops when body temperature falls below 24°C. Hypothermia can also markedly increase rate of apoptosis in cardiomyocytes [7].

## The influence of cold environment on the circulatory system

Sillesen *et al.*[8] revealed that injury-induced blood loss activates the coagulation system, thereby promoting the generation of blood clots to inhibit additional bleeding. Cold exposure can affect the coagulation cascade which ultimately damages platelet function. When body temperature declines to 32.8°C, the activity of coagulation factor IV can be reduced by up to 33%. Local tissue freezing significantly increases red blood cell numbers, hemoglobin, and capillary resistance, and then decreases the deformability of red blood cells, promotes leukocyte adhesion/activation, increases blood viscosity, and ultimately promotes the formation of thrombus. These changes can cause a vicious cycle leading to disturbances in the microcirculation of frostbite-affected tissue and eventually causing damage to the body [9]. Heart rate, respiratory rate, and blood pressure rise between normal and 32.2°C, but decrease under the 32.2°C; below 27.8°C the heart’s rhythm becomes dangerously disordered.

## The influence of cold environment on the respiratory system

The effects of cold exposure on the respiratory system include decreased respiratory cilia movement and increased respiratory secretions and viscosity [10]. Pulmonary edema may emerge if body temperature drops below 25°C. Respiratory reflex stimulation typically accelerates during the early phase of hypothermia, which is followed by reduced respiration rate and tidal volume. Severe conditions can inhibit the medullary respiratory center and weaken tracheal and bronchial cilia movement, thereby causing injury to the airways.

## The influence of cold environment on the urinary system

The early phase of hypothermia has a diuretic effect, which may be associated with peripheral vasoconstriction, inhibited antidiuretic hormone, and elevated central blood volume. However, if body temperature continues to drop below 32.2°C, both renal blood flow and glomerular filtration rate decrease, which, if severe, may cause acute renal failure [11].

## The influence of cold environment on metabolism

Low temperature could double the metabolic rate, which depletes energy reserves. If body temperature drops further, muscle tremors may occur. Muscle tremors boost the metabolic rate by 6-fold compared to the resting state; thus, energy exhaustion is more pronounced. A further decrease in body temperature results in decreased blood flow, disrupting electrolyte and acid–base balances eventually causing hypoxic liver injury.

## Current research status of the impact of cold environment on combat trauma treatment

In the pre-hospital environment, victims suffering from hypothermia in addition to moderate or severe brain injury caused by local, blunt trauma may show a 3-fold increase in mortality rate, and death may occur shortly after their arrival at the hospital. An examination of the Pennsylvania Interstate pre-hospital trauma database also revealed that injured patients aged 16 years or older displayed a similar rate of mortality if hypothermia developed. A retrospective analysis on 12-year burn patient data in New York showed that hypothermia is common among extensive burn patients, who also have a high mortality rate [12].

## Hypothermia is an independent risk factor for combat trauma-related death

It has been proposed that hypothermia is an independent physiological predictor of pre-hospital death rates [13, 14]. Arthurs *et al.* analyzed data from 2,848 patients who had been treated in a hospital for combat trauma, a sample in which 18% of the patients were hypothermic, and concluded that hypothermia was significantly correlated with the Glasgow Coma Scale (GCS), tachycardia, hypotension, lower hematocrit, and acidosis. If a victim is exposed in a cold environment, he or she may violently shiver and sustain a rapid dissipation of body heat via conduction, convection, and radiation; as a consequence, the ability to regulate core body temperature is lost. If body temperature drops below 32°C, mortality rate can reach 21%; furthermore, the same low body temperature along with bleeding caused by combat injury may result in a 100% death rate. Deaths caused by hypothermia are also associated with bleeding. Duan and colleagues*.*[15] argued that acute traumatic coagulopathy (ATC) is commonly seen among patients with severe injury and leads to uncontrolled bleeding diathesis, which is an important contributor to trauma death.

## Controversies

There are still a few scholars who oppose the designation of hypothermia as an independent risk factor for trauma death, arguing that hypothermic patients are often accompanied by more severe diseases. Lapostolle F *et al.*[16] believed that the key risk factor for the onset of hypothermia were the severity of injury, but other factors such as environmental conditions and the emergency medical services provided were also significant. Changes in practice could help reduce the impact of factors such as infusion fluid temperature and mobile unit temperature. Nevertheless, this opinion has been challenged. Bukur *et al.*[17] first classified injured patients according to severity, further divided each injury level into sub-groups of different body temperatures, and assessed their diseases. The authors found that the rate of mortality was significantly different between the hypothermic and non-hypothermic groups, indirectly arguing against the theory that disease severity, but not hypothermia, leads to a high mortality rate.

## The prevention of hypothermia in cold environments

Studies have revealed that in the early stages of combat trauma treatment, measures that effectively restore body temperature are imperative as they are conducive to improving the prognosis of combat trauma [18]. After the Korean War, the Afghanistan war represents another large-scale armed conflict in a cold region. A study was aimed to explore the extent of cold weather injuries in the military population [19]. 18,214 patients were involved in the study. 19 cases were identified cold-weather injury in the Afghanistan Conflict. Two cases of frostbite were identified with only 1 likely requiring surgical intervention. No cases occured in cold-weather injury in Iraq. The 19 cold-weather injuries were decreased greatly compared with the number of cold-weather injuries in the Korean War which is reported up to 6300 cases. This suggested that cold-weather injuries colud be prevented by the shorter and weather-dependent engagements, education about cold-weather, and improved equipment of the soliders.

## Common rewarming methods

A simple method to address the damaging effects of cold temperature is to employ passive rewarming strategies. For example, patients should be rapidly evacuated from the cold environment, re-warmed using an insulation blanket, and covered with a woolen blanket in the ambulance rather than being exposed to the air and losing body heat during examination of the injury. This is important because once hypothermia occurs, there is no easy way to recover body temperature. The above method is only applicable for moderate hypothermia and is ineffective for patients whose thermoregulatory mechanisms are impaired. Currently, Hypothermia Prevention Management Kits are used by the United States Army to keep patients warm during their transfer [20]. More invasive re-warming methods require special devices and training; thus, they are unsuitable for temperature restoration in pre-hospital environments. The typical methods of such rewarming techniques include body core rewarming and body peripheral rewarming, where the former outperforms the latter. In addition, rewarming devices can also be used for airway warming, transfusion warming, or as a cover for surface rewarming. These devices may also be installed on an emergency or evacuation platform for mandatory ventilation rewarming. Another effective method of temperature maintenance is to place an insulated heating package on the patient’s head, back, or armpit.

Under simulated battlefield conditions, the aforementioned methods reliably prevent hypothermia in animal models. Based on the Joint Theater Trauma System Clinical Practice Guideline (CPG), a study was designed to evaluate the effectiveness of CPG in treatment in the prevention of hypothermia-trauma complications. The results of this study revealed that the application of CPG can decrease the occurrence of hypothermia, where a woolen blanket is most frequently used during evacuation. Allen *et al.*[20] tested three active hypothermia prevention products, including the Hypothermia Prevention Management Kit (HPMK), Ready-Heat, and Bair Hugger, as well as five passive products (woolen blanket, space blanket, Blizzard blanket, human remains pouch, and Hot Pocket) [21]. Their results showed that the active products led to better outcomes than the passive methods, with HPMK showing the best results by maintaining significantly higher temperatures for 120 min. The study also showed that the active prevention devices did not reach 44°C, a temperature which damages human tissue, after 6 hours use. The best-performing passive products were the Hot Pocket and Blizzard blanket, which were able to sustain high temperatures for 120 min. Hence, all of the active devices and most of the passive tools were superior to a woolen blanket. Under conditions close to room temperature, the Hot Pocket, Blizzard blanket, and other active devices were as effective as HPMK. Of course, ensuring that there is insulation between the victim and the ground or the stretcher is a top priority because the body can lose heat through conduction. Attention should also be paid to keeping the head and feet warm, as these areas lose most of the body heat. Lastly, it is important to note that the venous infusion of fluid at room temperature may rapidly decrease body temperature; therefore, fluids need to be warmed to at least 37.8°C, or for hypothermic patients, 40.0-42.2°C.

## The treatment of hypothermia in cold environments

### The application of blood products in the coagulation disorders treatment

The currently acknowledged lethal triad is comprised of hypothermia, metabolic acidosis, and coagulopathy [22]. Hypothermia usually comes together with and may aggravate acidosis and coagulopathy, which again may be associated with high mortality [1], especially in cold regions. Any one of these factors may lead to death if not properly treated. Coagulopathy resulting from severe trauma complicates the control of bleeding and therefore increases the rate of mortality in combat-wounded victims. Hypothermia can lead to coagulation disorders, which increase the incidence of bleeding and “non-fatal triad” as is in the non-cold regions. Take an experimental study for example [23], 40 Sprague–Dawley rats were used to established a model for uncontrolled bleeding by being exposed to a femoral artery injury, and allocated to normal temperature group or hypothermia group. The hypothermic group was cooled to 30°C and then rewarmed after 90 min. The volume of bleeding was recorded. There was no difference between groups during the initial bleeding. These rebleeding volumes accounted for about 41% of total blood volume in the hypothermic group, while the corresponding figure for the normothermic rebleeders was only 3% of blood volume (*P* < 0.001). This study demonstrated that the risk of rebleeding from a femoral injury is greater in the presence of hypothermia and suggests that factors other than temperature-induced coagulopathy also contributed to the increased hemorrhage.

Shock promotes the initiation of early coagulation pathways in which damaged tissues release thrombus-like substances and activate anticoagulation systems; coagulopathy is closely linked to subsequent treatments for acidosis, hypothermia, and hemodilution [24]. The prompt treatment of hypothermia is needed to correct the coagulopathy, thereby avoiding the occurrence of catastrophic and fatal disseminated intravascular coagulation (DIC). Methods to treat coagulopathy include infusion of recombinant activated factor VII (rFVIIa) or fresh whole blood. In addition, treatment with tranexamic acid (TXA) can decrease the mortality of patients who have been hospitalized for > 30d. Treatment of combat-wounded victims at the 31st Combat Support Hospital (31st CSH) in Baghdad between 2004–2005 revealed that there were some advantages to using fresh whole blood at 20-24°C; military doctors proposed that whole blood should be used under extreme conditions with low temperatures. Farrugia *et al.*[25] propose that in large-volume hemorrhage, fresh whole blood in may be superior to whole blood that has been reconstituted from multiple individual components. Nevertheless, research conducted by the United States Army revealed that between March 2003 and July 2007, over 6,000 units of warm fresh whole blood were used for transfusion in field hospitals of Afghanistan and Iraq. By December 2008, 20 patients became infected with hepatitis because of whole blood transfusion [26]. As a consequence, the United States Army Medical Department issued an order that fresh whole blood can only be used if there is no other blood source.

### Controlled hypothermia

Damage control resuscitation is a standard pre-hospital treatment method in field hospitals. With a basic strategy of low pressure, low capacity, delaying, and low temperature, this technique emphasizes the principles of field medicine: “forward-looking and strengthening, having advanced equipment, and focusing on emergency.” In this way, medical staff can provide advanced trauma support for combat treatment. The invention and application of respiratory and circulatory support technologies developed by the United States Army during the Gulf War are the most prominent breakthrough in combat injury treatment. Such therapeutically low temperatures are different than those induced in cold environments, constituting an active method to achieve controlled low temperature; by adjusting fluid resuscitation to a lower than normal body temperature, this technique extends the ideal treatment window for hemorrhagic patients to receive life-saving hemostasis and resuscitation.

Baron Dominique-Jean Larrey (1766–1842) was the first to propose hypothermia as a therapeutic method [27]. To test the feasibility of the cardiopulmonary bypass/extracorporeal membrane oxygenation (CPB/ECMO) system which was designed at the Cleveland Clinic Foundation’s (CCF), a study was conducted by a porcine model mimics frontline combat wounds [28]. The animals were induced with lethal massive hemorrhage of the major blood vessels and underwent repair of the blood vessels under a state of deep hypothermic arrest by CCF. The animals survivedt and were monitored for neurological conditions, cognitive function, the damage of vital organs, and complications after 3 weeks. 83% of the test animals survived. and there was no significant difference in neurological impact, organ dysfunction, or complication occurrence between the treatment outcomes and the conventional equipment. Therefore, the CCF/CPB/ECMO system can be employed to facilitate the repair of lethal major vascular injuries through deep hypothermia, especially for those wounded under harsh conditions.

Furthermore, hypothermia therapy is also used for cerebral hypoperfusion after cardiopulmonary resuscitation in clinical practice. For instance, Pacini *et al*. [29] pointed out that antegrade selective cerebral perfusion (ASCP) with moderate hypothermia at 26°C provides good brain protection during aortic arch surgery. Moreover, during circulatory arrest, moderate hypothermia also offers good protection of visceral organs within the limited periods (< 60 min) of visceral ischaemia, by the means of reducing the systemic inflammatory response and the reperfusion organ injury. It was also suggested that ASCP) and mild systemic hypothermic circulatory arrest can be safely applied to complex aortic arch surgery even in a subgroup of patients with up to 90 minutes of antegrade cerebral perfusion (ACP) [30]. Unilateral ACP might be advantageous than bilateral ACP in that it reduces the incidence of embolism arising from surgical manipulation on the arch vessels. Zierer *et al.*[31] believed that unilateral ACP in a pressure-controlled manner during mild systemic hypothermia is a safe protection strategy in elective aortic arch surgery.

Taken together, the current treatment and prevention measures for combat trauma in cold environments remain open for improvement. In the future, molecular techniques are needed to elucidate the mechanisms underlying body injury due to the cold environment, including the study of cell factors associated with cold trauma and the further exploration of relevant prevention methods.

## Abbreviations

ACP: Antegrade cerebral perfusion
ASCP: Antegrade selective cerebral perfusion
ATC: Acute traumatic coagulopathy
CCF: Cleveland Clinic Foundation’s
CPB/ECMO: Cardiopulmonary bypass/extracorporeal membrane oxygenation
CPG: Clinical practice guideline
CSH: Combat Support Hospital
DIC: Disseminated intravascular coagulation
GCS: Glasgow coma scale
HPMK: Hypothermia prevention management kit
PLA: People’s Liberation Army
rFVIIa: Recombinant activated factor VII
TXA: Tranexamic acid.
